# Compartmentalization of the gut viral reservoir in HIV-1 infected patients

**DOI:** 10.1186/1742-4690-4-87

**Published:** 2007-12-04

**Authors:** Guido van Marle, M John Gill, Dione Kolodka, Leah McManus, Tannika Grant, Deirdre L Church

**Affiliations:** 1 Department of Microbiology and Infectious Diseases, University of Calgary, Calgary, Alberta, Canada; 2 Department of Pathology and Laboratory Medicine, University of Calgary, Calgary Alberta, Canada; 3 Division of Microbiology, Calgary Laboratory Services, Calgary, Alberta, Canada

## Abstract

**Background:**

Recently there has been an increasing interest and appreciation for the gut as both a viral reservoir as well as an important host-pathogen interface in human immunodefiency virus type 1 (HIV-1) infection. The gut associated lymphoid tissue (GALT) is the largest lymphoid organ infected by HIV-1. In this study we examined if different HIV-1 quasispecies are found in different parts of the gut of HIV-1 infected individuals.

**Results:**

Gut biopsies (esophagus, stomach, duodenum and colorectum) were obtained from eight HIV-1 infected preHAART (highly active antiretroviral therapy) patients. HIV-1 Nef and Reverse transcriptase (RT) encoding sequences were obtained through nested PCR amplification from DNA isolated from the gut biopsy tissues. The PCR fragments were cloned and sequenced. The resulting sequences were subjected to various phylogenetic analyses. Expression of the *nef *gene and viral RNA in the different gut tissues was determined using real-time RT-PCR. Phylogenetic analysis of the Nef protein-encoding region revealed compartmentalization of viral replication in the gut within patients. Viral diversity in both the Nef and RT encoding region varied in different parts of the gut. Moreover, increased *nef *gene expression (p < 0.05) and higher levels of viral genome were observed in the colorectum (p < 0.05). These differences could reflect an adaptation of HIV-1 to the various tissues.

**Conclusion:**

Our results indicated that different HIV-1 quasispecies populate different parts of the gut, and that viral replication in the gut is compartmentalized. These observations underscore the importance of the gut as a host-pathogen interface in HIV-1 infection.

## Introduction

Recently there has been an increasing interest and appreciation for the gut as a viral reservoir and an important host-pathogen interface in human immunodefiency virus type 1 (HIV-1) infection [1-4]. The gut associated lymphoid tissue (GALT) is the largest lymphoid organ infected by HIV-1. Studies on simian immunodeficiency virus (SIV) have indicated the gut is an important site for CD4^+ ^T-cell depletion [1,4], and this appears to be similar in humans [5]. The inflammatory milieu in the gut is considered to play a key role in CD4^+ ^cell loss, as a lack of CD4^+ ^cell replenishment in the gut of HAART treated HIV-1 infected individuals was associated with increased inflammatory gene expression and immune activation [2]. These observations have also led to the hypothesis that HIV-1 may "hide" from antiretroviral therapy in the gut [2]. This would be consistent with the notion that the gut could act as a separate reservoir for viral replication [6]. However, very little is known about the HIV-1 quasispecies that reside in the gut.

Viral variability significantly affects pathogenesis and infection. Disease progression in HIV-1 infection is accompanied by an increasing diversity in viral sequences found within the infected individual [7]. HIV-1 is highly divergent due to the error-prone reverse transcription step in the HIV-1 life cycle [8]. Factors such as viral fitness, availability of target cells for infection, antiretroviral therapy, duration of infection and the host immune response influence which viral quasispecies arise during the course of infection [9-13]. For both SIV and HIV-1 it has been suggested that the immune system can push viral evolution towards HIV-1 quasispecies with increased pathogenic properties [7,14-17]. In HIV/AIDS patients on antiretroviral therapy, viral sequences evolved over time in genes not targeted by the drugs, despite undetectable plasma viral loads [18-20]. These observations suggested that viral replication continued in tissues or cell compartments not efficiently targeted by the antiretroviral drugs. The contribution of the gut to increasing viral diversity in the host is unresolved. In addition, it is unclear to what extent viral replication in the gut is compartmentalized. The distribution and composition of the lymphoid tissues vary amongst different locations in the gut. For instance, lymphocytes in the small intestine can be found in organized structures, so-called Peyer's patches, but are also found in the lamina propria and as intraepithelial lymphocytes throughout the gut (reviewed in [21]). The Peyer's patches are found in the duodenum, but their frequency increases further down the small intestine, with the largest number present in the ileum (reviewed in [22]). In humans, lymphocytes in the large intestine (i.e. cecum, colon and rectum) are found as isolated lymphoid follicles, with the highest frequency in the rectum [23,24]. The distribution of the type of T-cells in the GALT is different than the periphery, as 70% of the intraepithial lymphocytes in the small intestine are CD8^+ ^T-cells [25]. Moreover, throughout the intestine the majority of CD4^+ ^T-cells are CCR5 positive [26]. Therefore, the different parts of the gut could select for different HIV-1 quasispecies, and thus act as reservoirs for different viral strains.

The goal of the current study was to determine if viral replication in the gut is compartmentalized. We analyzed HIV-1 sequences of the Nef (negative factor) and the reverse transcriptase (RT) encoding region amplified by PCR from biopsy material taken from different locations within the gut of HIV-1 infected individuals. These analyses demonstrated that for both RT and the Nef encoding region viral diversity differed significantly among the various gut tissues, and phylogenetic analyses clearly demonstrated clustering of *nef *DNA sequences at different sites. Furthermore, our observations suggested compartmentalization of HIV-1 replication in different parts of the gut, and indicated that the gut is a distinct multi-compartment viral reservoir in HIV-1 infected individuals.

## Results

### Clustering of HIV-1 *nef *sequences by gut tissue compartment

To get insight into potential compartmentalization of HIV-1 replication at different locations within the gut, we focused on analyzing the Nef and RT encoding regions of HIV-1. These regions were amplified by nested PCR from DNA isolated from different gut tissues (esophagus, stomach, duodenum and colorectum) and peripheral blood lymphocytes (PBL). The samples were obtained from a previously described cohort of HIV seropositive homosexual men followed at the Southern Alberta Clinic (SAC), Calgary, Alberta, from 1993 to 1996 [6,27]. This cohort was recruited prior to the introduction of HAART (Highly Active Antiretroviral therapy) at the SAC in late 1997. Eight patients at various clinical stages of HIV infection/AIDS were selected and gut tissue samples from one visit were analyzed (Table 1). Although cDNA was synthesized and viral sequences could be detected with our real-time RT-PCR analysis using small amplicons (discussed in sections below), the sequences spanning the entire viral regions of interest were the most readily and consistently amplified from DNA. Therefore, we focused on these proviral sequences for the current study. Analysis of these sequences also exploits the "banking-effect" of proviral DNA in the chromosomal DNA of different cell populations offering some insight into "the history" of the infection [28] and seeding of the gut tissues. We chose to analyze the HIV-1 Nef and RT protein encoding sequences as these proteins have various effects on viral replication and the RT region has been shown to evolve by tissue compartment [8,29-31]. Both proteins are targeted by the cellular immune response [32-34], and therefore suitable targets to determine how HIV-1 evolves in different parts of the gut. The Nef protein is highly variable [32], but is relatively more conserved than the highly diverse envelope protein [35], which could make it easier to detect phylogenetic relationships within the patient population. Finally, the Nef protein has been implicated as an important pathogenic determinant of HIV-1 [36-47], and its analysis could shed some light on the evolution of pathogenic HIV-1 strains in the gut.

**Table 1:** Patients

**Patient**	Viral load Log(copies)/mL*	**CD4**^+ ^Cells/mL*	Antiretroviral Drugs*
#1	2.7	264	ddI, AZT
#2	5.8	18	None
#3	5.6	77	AZT
#7	4.5	146	AZT
#8	4.4	325	ddI
#19	3.9	77	ddI
#42	5.6	9	None
#60	4.5	48	AZT

* Viral loads, CD4^+ ^counts and antiretroviral therapy at time biopsies were collected

**Figure 1:**
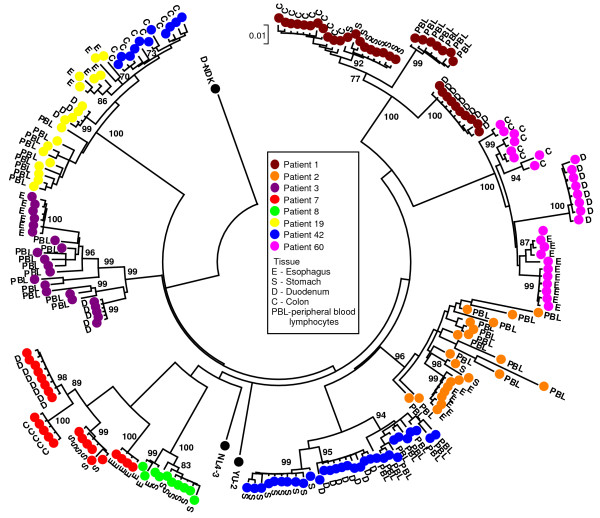
**Bootstrap Neighbor-Joining tree of the sequences of the Nef encoding region obtained from gut tissues**. Nef sequences clustered by individual patients (indicated by colors). Closer examination of these sequences revealed clustering of Nef sequence by tissue compartment (esophagus (E), stomach (S), duodenum (D), colorectum (C) and PBL) within patients, indicative of compartmentalization of viral replication in the gut, resulting in the evolution of different HIV-1 quasispecies in different parts of the gut. (Bootstrap values > 70 are indicated.)

**Figure 2:**
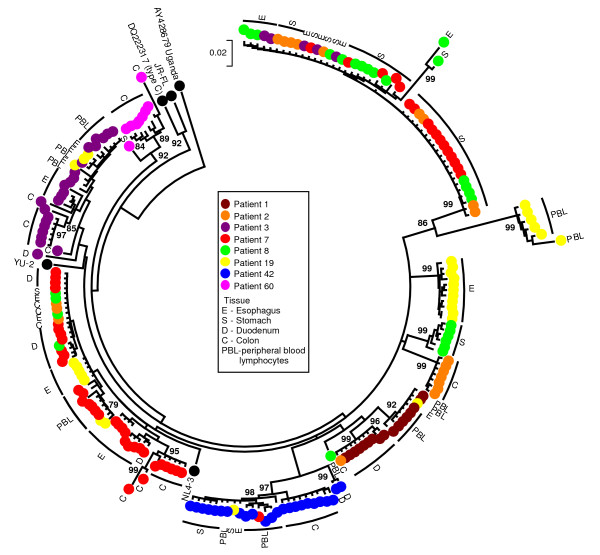
**Bootstrap Neighbor-Joining tree of RT encoding sequences obtained from gut tissues**. Clustering was observed of RT encoding sequences by patient and tissue but not to the same extent as observed for the Nef encoding sequences. Closer examination of the tree revealed clustering of a large number of sequences derived from the esophagus and stomach from different patients, suggesting some selection for esophagus and stomach specific RT encoding sequences. (Bootstrap values > 70 are indicated.)

HIV-1 viral sequences were amplified from PBL and biopsy tissue DNA using our nested PCR protocol. For seven patients RT and Nef encoding sequences were obtained from 3 or more tissues (gut tissues and/or PBL), while for one patient (#8) only sequences from two tissues could be obtained (stomach and esophagus). Neighbour-Joining trees revealed clustering of the *nef *sequences by individual patient (bootstrap values of >90) (Fig. 1). The clones of the Nef encoding sequences also clustered by gut tissue from which they were obtained (i.e. esophagus, stomach, duodenum and colorectum). No mixed clustering with sequences of different tissues was observed, indicating that within a patient distinct HIV-1 quasispecies were found within different parts of the gut. Analysis of the clustering pattern for the patients for which we were able to obtain viral sequences from PBL revealed clustering of these sequences with each other (bootstrap value >90) (Fig. 1). This indicated that the viral quasispecies found in the periphery were different from those found in the gut. No obvious phylogenetic relationship of PBL sequences with sequences of a particular gut tissue (i.e. esophagus, stomach, duodenum or colon) was observed among the different patients. In contrast to the Nef encoding region, similar tight clustering for the RT encoding region was not found for any of the patients (Fig. 2). However, for various patients a large number of the RT encoding sequences from the esophagus and stomach clustered together. The latter could suggest that there may be a selection for a particular RT encoding sequence in these tissues among patients.

**Figure 3:**
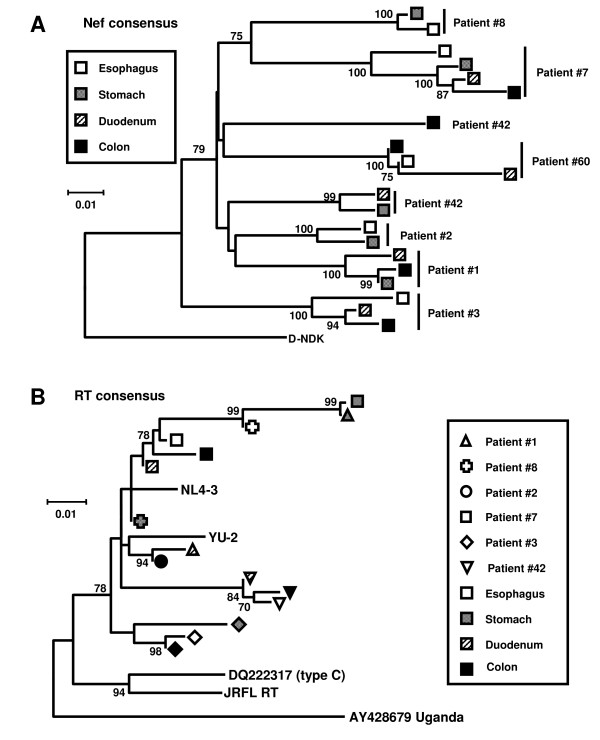
**Neighbor-Joining tree of the consensus sequences of the Nef and RT encoding region from gut tissues**. While no obvious clustering was observed for the RT **(B)**, Nef encoding sequences clustered by individual patients **(A)**. In patients 3 and 7 further clustering of sequences by upper (esophagus and stomach) or lower (duodenum and colorectum) gut tissues was observed. (Bootstrap values > 70 are indicated).

**Figure 4:**
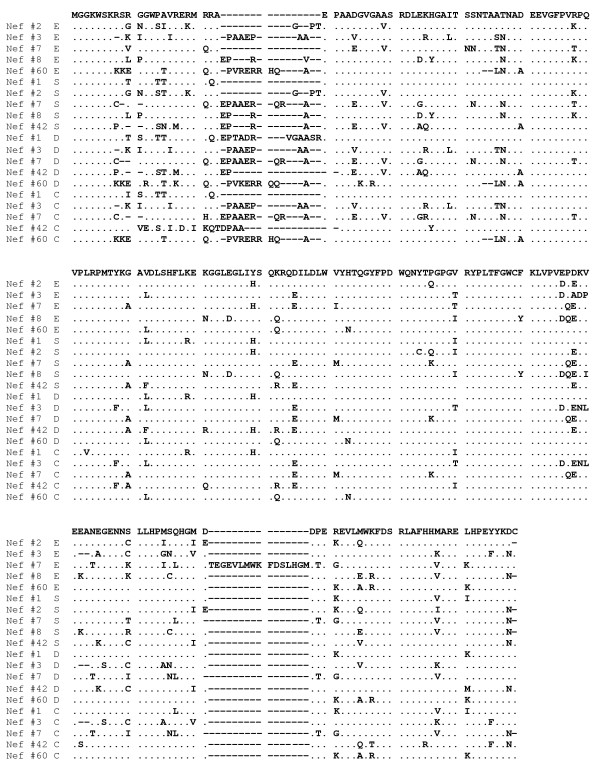
**Consensus Nef protein sequences for gut tissues of HIV-1 patients**. Consensus Nef protein sequences were obtained for the esophagus (E), stomach (S), duodenum (D), and colorectum (C). No specific signature sequences were observed for any of the gut tissues.

To corroborate our observations, the sequences from all clones for the Nef and the RT encoding region were used to derive consensus sequences for each tissue compartment for each patient. Bootstrap analysis of these consensus sequences revealed clustering of *nef *sequences by patient, and for two patients by upper (esophagus and stomach) and lower (duodenum and colorectum) gut tissue compartment (Fig. 3A) (bootstrap value >90). This suggested a clustering of *nef *sequences by upper or lower GI-tract in select patients. Similar to our previous results, clustering was again not observed for the RT encoding region (Fig. 3B). Analysis of the consensus Nef protein sequences obtained (Fig. 4) did not reveal any particular signature sequences for specific gut tissues. Taken together, these observations indicated that viral replication in the gut was compartmentalized, resulting in different HIV-1 quasispecies populating different parts of the gut.

### HIV-1 diversity in different gut tissues

**Figure 5:**
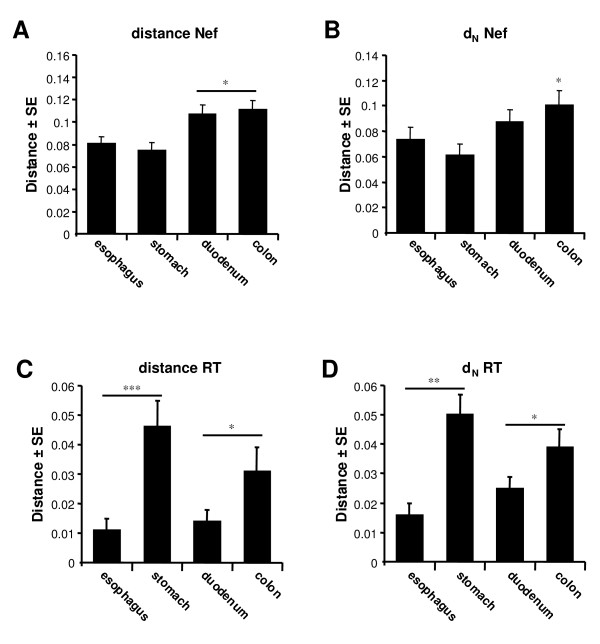
**Viral molecular diversity of the Nef encoding region in gut tissues of HIV-1 patients**. Viral Nef sequences were more diverse (higher mean total distance (d)) for the duodenum and colon compared to the stomach and esophagus **(A)**. Moreover, viral evolution tended towards a more diverse Nef protein in the colorectum as reflected by a significantly higher mean total non-synonomous distance (d_N_, i.e. amino acid changing mutations) **(B)**. A similar analysis of the RT coding region of HIV-1, also revealed significant differences in viral molecular diversity in the different tissues for both mean total distance (d) **(C) **and non-synonomous distance (d_N_) **(D)**. These observations indicated that different selection pressures were acting in different parts of the gut depending on the viral region. (* = *p *< 0.05, ** = *p *< 0.01 ***, = *p *< 0.001, Dunn's multiple comparison test).

To determine to what extent viral diversity differed among the different gut tissues, the mean total (d), and nonsynonymous (d_N_) pair-wise distances were calculated for the Nef and RT encoding sequences obtained from the esophagus, stomach, duodenum and colorectum tissues of all patients (Fig. 5). Significantly lower d and d_N _values (i.e. codon/amino acid changing substitutions) were observed for the RT encoding region for both the esophagus and the duodenum, compared to the stomach and colorectum (p < 0.001 and p < 0.05, respectively). In contrast, for the Nef encoding region, a significantly higher d value was observed in both duodenum and colorectum (p < 0.05). Further analysis of the Nef encoding region revealed a higher d_N _value in the colorectum (p < 0.05), suggestive of a more diverse Nef protein in the colorectum. Similar to the analysis of the consensus Nef protein sequences (Fig. 4), analysis of all the inferred protein sequences for both the Nef and RT protein obtained from the different patients did not reveal any signature sequences for any of the tissue compartments, nor were there any obvious differences in domains important for protein function [36-39,48-50](data not shown).

### Increased viral replication and *nef *gene expression in the colorectum of HIV-1 infected patients

**Figure 6:**
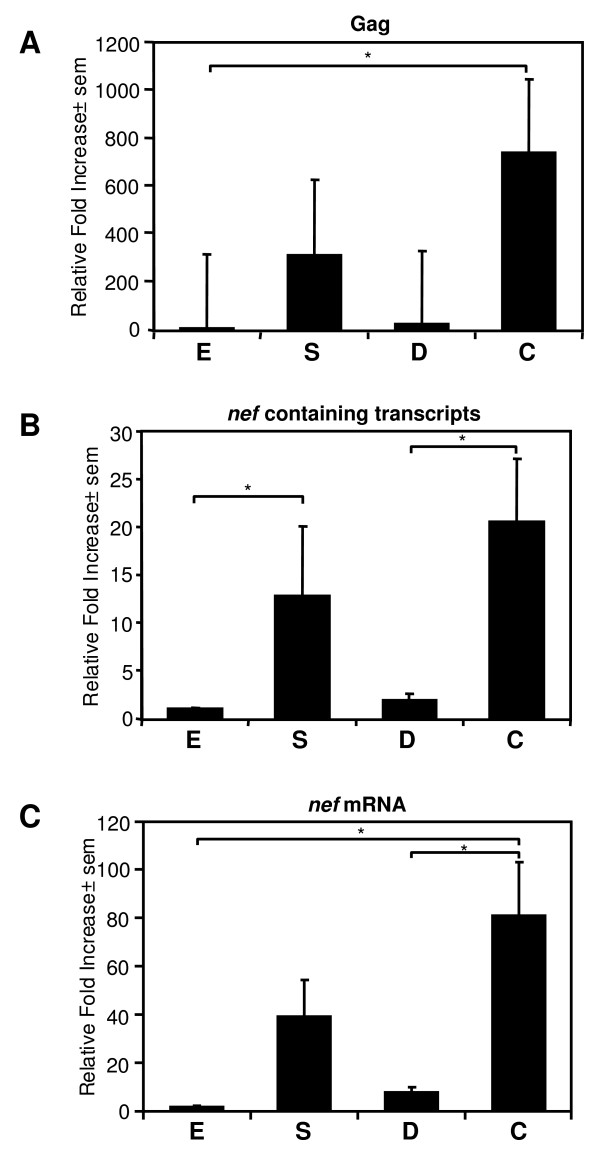
**Real-time RT-PCR analysis of viral expression in gut tissues. (A) **Real-time RT-PCR analysis of *gag *gene expression levels (viral genomic RNA normalized against GAPDH mRNA levels) in the esophagus (E), stomach (S), duodenum (D) and colorectum (C) tissue of HIV-1 infected individuals. Increased viral *gag *gene (viral genomic transcripts) expression in the colorectal tissues compared to the esophagus were observed. All *gag *expression levels were expressed relative to the *gag *expression levels in the esophagus (* = *p *< 0.05, Dunn's multiple comparison test). Real-time RT-PCR analysis of all viral RNAs (genomic as well as viral mRNA transcripts) containing the Nef protein open reading frame **(B) **and *nef *gene specific mRNA expression levels **(C) **(normalized against GAPDH mRNA levels) in the esophagus (E), stomach (S), duodenum (D) and colorectum (C) tissue of HIV-1 infected individuals. Differing RNA expression levels with increased viral *nef *gene expression in the colorectal tissues were observed. Again all RNA expression levels were expressed relative to the RNA expression levels in the esophagus (* = *p *< 0.05, Dunn's multiple comparison test).

The differences in viral diversity suggested that HIV-1 evolved to varying degrees in the different gut tissues. A previous report observed differences in viral loads between blood and colorectum [51], indicative of differences in viral growth between these compartments. To extend the observations obtained with our phylogenetic analysis, we used real-time RT-PCR to determine the relative levels of viral genomic RNA in our patients in esophagus, stomach, duodenum and colorectum (Fig. 6A). The fold increase in transcript levels was expressed relative to the levels observed in the esophagus, since in all patients we found the lowest level of transcript in this tissue. Significantly higher levels of viral genomic RNA in the colorectum compared to esophagus were observed in the 8 patients analyzed in this study (p < 0.05), suggesting HIV-1 replication differs in different parts of the gut. Finally, as our results indicated a more diverse Nef protein in the colorectum, we used real-time RT-PCR to analyze the expression of all HIV-1 RNA transcripts (genomic and mRNAs) containing the Nef protein open reading frame (Fig. 6B), as well as *nef *gene specific mRNA transcripts [52](Fig. 6C) in the different gut tissues. Indeed, an increased expression of viral mRNA and *nef *gene specific mRNA transcripts (p < 0.05) was observed among all patients in the colorectum.

## Discussion

The current study clearly indicated that different genes of HIV-1 evolved differently in different parts of the gut. Previous studies have shown that HIV-1 quasispecies found within a patient in the colorectum were different from those found in blood and brain [29,53], suggesting that gut is a separate evolving compartment for HIV-1 replication. To our knowledge our study is the first demonstration that HIV-1 replication in the gut is in itself further compartmentalized. Distinct viral quasispecies were found in the esophagus, stomach, duodenum and colorectum that were different from those found in the periphery (PBL). The data also indicated that viral replication and viral *nef *gene expression, varied across the gut tissues. The results obtained for the Nef protein encoding region in our cohort is the most convincing evidence of compartmentalization of HIV-1 replication, resulting in the evolution of different HIV-1 quasispecies in different parts of the gut.

Varying viral diversity was observed for both the Nef and the reverse transciptase (RT) encoding region in the gut. Both regions are primarily targeted by the cellular immune response, which will significantly impact viral quasispecies evolution [32-34]. However, their role in determining viral replication and infectivity could also shape viral evolution in these different gut tissue compartments [8,29-31]. The RT region did not exhibit the same pattern of clearly defined clustering that was observed for the Nef encoding region. As the RT region is highly conserved due to its importance in viral replication, distinct clustering would be less likely to be picked up in these phylogenetic analyses due to high levels of sequence homology. However, despite the lack of clustering of the RT region, we did observe a consistent clustering pattern of a large number of stomach and esophagus derived sequences from different patients. This could suggest that there are particular requirements for the RT protein for the infection of the esophageal and stomach tissues, but we did not observe distinct RT protein sequence motifs. Alternatively, the immune system could have selected for specific RT protein sequences in these tissues. This is consistent with compartmentalization of the infection of the gut by HIV-1, and again illustrated that the RT is under different selection pressures compared to the Nef encoding region.

Of particular interest was the more diverse *nef *gene in the colorectum among the patients. Differences in immune selection pressures in the colorectum could push viral evolution towards more diverse *nef *sequences. The Nef protein plays a role in determining HIV-1 infectivity and viral replication [30,31]. The observed differences could reflect different adaptation of HIV-1 to the colorectal tissues, which may explain the higher viral RNA and the increased *nef *gene expression in the colorectum, further supporting compartmentalization of HIV-1 replication in the gut. Alternatively, the differences in viral replication in the different gut tissues could be the result of different levels of infection due to differences in the amount of infectable cells (i.e. CD4^+ ^cells) in those tissues, or differences in the amount of viral RNA produced by each infected cell. In turn, the elevated viral replication could result in higher viral diversity in the colorectum due to increased error prone replication. Of note, in the brain all these factors not only affect viral evolution and compartmentalization of viral replication, but also play an important role in pathogenesis (reviewed in [54]).

The patients analyzed in the current study were primarily chosen based on their HIV/AIDS status and not selected on any other common pathological features. In addition, as our sequences were derived from DNA, we did not sample the viruses that were actively replicating and responsible for pathogenesis in the different tissues. This may explain why we did not find any clear differences in sequence motifs in the Nef protein that could be linked to viral pathogenesis or altered protein function [36-39]. Alternatively, the lack of common features among the Nef protein sequences may be due to the fact that in specific patients the Nef protein may play a major role in pathogenesis, while in others it may not. More patients will need to be analyzed to address this question. Nonetheless, our results clearly indicated that different HIV-1 strains end up in the different tissues. This could be the result of particular tissue requirements for HIV-1, immune selection, as well as the earlier mentioned differences in the number of HIV-1 infectable cells in these tissues. The latter may also be reflected by the clustering of Nef protein encoding sequences by upper and lower GI tissues in some of the patients. The immune response and the cells of the upper or lower GI tissues may have distinct features in common, thereby selecting for more related HIV-1 varieties. However, as we only observed this clearly in two of the patients analyzed, this may not be a common feature. Again, the implications of these observations for HIV-1 pathogenesis remain to be determined.

Despite the fact we did not find direct links to pathogenesis in our analyses, the clustering and differing viral diversity of the Nef protein encoding sequences is of interest. The Nef protein plays many roles in pathogenesis, which is underscored by the observation that deletions in the *nef *gene, rendering the protein nonfunctional, have been associated with long-term non-progression or absence of HIV-1 associated neurological disease [40-47]. Indeed, the Nef protein has many cytotoxic properties[37-39,55-61]. It has both apoptotic and anti-apoptotic activities, and also has various effects on the infected cell (reviewed in [47,55-57]). The Nef protein also has proinflammatory actions and its expression results in the induction of cytokines and chemokines, which is affected by the Nef protein sequence [62-64]. Given the important pathogenic role proposed for gut mucosal inflammation in CD4^+ ^cell depletion, [1,2,4,5], our observations may also point to a pathogenic role for the Nef protein in the gut. This notion is strengthened by previous observations in HIV/AIDS patients with neurological disease in which increased viral diversity in blood and brain was associated with neurological impairment [14]. The reverse is also true, and we have shown that for the Nef encoding region, viral evolution tended towards a more conserved and possible more pathogenic Nef protein in the brain [62]. These and other observations indicate that host-dependent selection pressures can push viral evolution towards viral strains with a more pathogenic phenotype, which is relevant for both systemic and organ specific pathogenesis [7,14-16]. The increased viral diversity of the Nef protein encoding region in the colorectum may be the result of increased viral replication. This could increase the chance of pathogenic HIV-1 strains evolving in this part of the gut. Further studies involving patients categorized by pathology will be required to determine to what extent this plays a role in HIV-1 pathogenesis, and are currently ongoing in the laboratory.

Finally, it has been proposed that HIV-1 can "hide" in the gut from antiretroviral therapy [2]. It is also possible that the gut could act as a reservoir for pathogenic viral strains that are not easily identified in the periphery, in that respect acting as a "hide out". Our results showed that different HIV-1 quasispecies were found in the gut tissues that differed from those found in the PBL within each patient, which would be consistent with this notion. However, analysis of multiple viral regions of the actively replicating viruses in the different tissues over multiple visits will be necessary. These studies will allow us to determine how the gut is seeded, and if the different gut tissues not only act as "hide-outs" for HIV-1 drug resistant strains, but also as reservoirs for pathogenic viral strains.

## Conclusion

In conclusion, our observations indicate that the different parts of gut act as distinct compartments for HIV-1 replication containing different HIV-1 quasispecies. These results suggest that the gut could contribute to overall viral diversity. Together with the important role the gut plays as host-pathogen interface in the development of AIDS, this has major implications for treatment of this devastating disease. The complex nature of the gut viral reservoirs has to be taken into account when designing therapeutic approaches, as the gut may be a sanctuary site for drug resistant strains or a source of pathogenic HIV-1 strains.

## Materials and methods

### Patients

Patients were enrolled from a previously described cohort of HIV seropositive homosexual men followed at the Southern Alberta Clinic (SAC), Calgary, Alberta, from 1993 to 1996 [6,27]. This study was reviewed and approved by the Office of Medical Bioethics of the University of Calgary and all patients signed an informed consent at enrollment. Patients were prospectively followed and laboratory testing included plasma viral load and CD4 counts, for each patient during each visit. In addition upper and lower endoscopies were performed in order to harvest tissue for further testing. This cohort was recruited prior to the introduction of HAART (Highly Active Antiretroviral therapy) at the SAC in late 1997. Antiviral therapies during the study consisted of no treatment, monotreatments with AZT, DDI, DDC, D4T, and 3TC or combinations thereof.

### Gut biopsy samples

Gastrointestinal tract biopsies were taken during the study period at the level of the esophagus, stomach, duodenum and colorectum. The biopsies taken via endoscopic procedure were shipped cryopreserved and stored at -70°C, within 1 hour of collection [6]. Plasma or serum was collected and stored at -70°C and peripheral blood lymphocytes (PBL) were isolated and stored in liquid nitrogen[6,27]. Eight patients at various clinical stages of HIV infection/AIDS were selected. All patients had a CD4 count of lower than 400 at the time of biopsy (Table 1). Nucleic acid sequence analyses were performed on the gut biopsies taken at one visit for this subset of patients.

### Nucleic acid isolation and PCR amplification of viral sequence from PBL and gut biopsies

Chromosomal DNA was isolated from the tissue biopsies from each patient using Trizol Reagent (Invitrogen, Burlington, ON). The HIV-1 proviral DNA regions encoding the Nef (negative factor) and RT (reverse transcriptase) protein were amplified using a nested PCR and 0.2 μg of chromosomal DNA as template. The nested PCR protocol for both the first and second round PCR, consisted of an initial denaturation step of 5 min at 94°C, 45 cycles of 1 min at 95°C, 1 min at the annealing temperature of the primer set used, 2 min at 72°C, followed by a final extension step of 10 min at 72°C. For the *nef *region, the primers used for the first round PCR were NEF8678F 5'-GTA GCT GAG GGG ACA GAT AG-3' and NEF9540R 5'-AGG CTC AGA TCT GGT GTA AC-3' (T_m _= 50°C), and for the second round NEF8748F 5'-GAA GAA TAA GAC AGG GCT-3' and NEF9425R 5'-AGT CCC CAG CGG AAA GTC CC-3' (T_m _= 40°C) [62]. For the RT region, the first round primers consisted of RT2470 5'-GTA CAG TAT TAG GAC CTA CAC CTG-3' and RT3261 5'-ATC AGG ATG GAG TTC ATA ACC CAT CCA-3' (T_m _= 55°C), and for the second round consisted of RT2604 5'-CCA AAA GTT AAA CAA TGG CCA TTG ACA-3' and RT3251 5'-AGT TCA TAA CCC ATC CAA AG-3' (T_m _= 55°C). All viral sequences were amplified using *pfu *polymerase to reduce introduction of mutations during amplification. In addition, dilution experiments of the template were performed to prevent selective amplification of the most dominant viral sequences at the expense of less frequent viral sequences, and thus skewing sampling of the viral quasispecies. For these experiments, 2 to 10 fold dilutions of the template DNA were made. The dilutions at which still an abundant PCR product could be obtained were used for our sequence analysis.

### Sequence and phylogenetic analysis

The resulting Nef and RT encoding PCR fragments were isolated from gel using the Qiaquick gel extraction kit (Qiagen Inc., Mississauga, ON) and directly sequenced to obtain overall consensus sequences for the different tissue compartments. In addition, the gel purified PCR fragment were cloned by filling in the incomplete fragment ends with Klenow, phosphorylation using T4 polynucleotide kinase, and insertion into the *Eco*RV site of pSL1180. All enzymes were obtained from Invitrogen (Burlington, ON) and New England Biolabs (Pickering, ON) and used according to the manufacturer's specifications. Where possible up to five to ten clones per patient/sample were analyzed to determine the composition of the viral quasispecies for the Nef and RT encoding region. DNA sequences were determined by automated sequencing on an ABI sequencer (Applied Biosystems, Streetsville, ON) and a Li-Cor 4300 DNA Analysis sequencing system (Li-cor Biosciences, Lincoln, NE) according to the manufacturers' protocols and reagents. All sequences have been submitted to Genbank (accession numbers EF656787 to EF657121)

The inferred amino acid sequence for each cloned nucleic acid fragment was obtained for each sample. These protein sequences were screened for the integrity of the coding sequences (i.e. stop codons, deletions and/or insertions), as well as common amino acid motifs between the different patients and samples. In addition, changes in amino acid residues or motifs that have been shown to be important for function of the Nef and RT protein were analyzed [36-39,48-50]. The nucleic acid sequences were subjected to an extensive phylogenetic analysis, using the MEGA version 3.1 software package [65]. Neighbor-Joining trees were constructed using the Kimura-2-parameter model with 5000 replicates for the bootstrap analysis. Bootstrap values of >70 were considered significant. Reference sequences of prototypic HIV isolates were included to rule out possible contamination with laboratory strains. The MEGA software was also used to calculate mean total (d), nonsynonymous (d_N_, i.e. codon changing substitutions) and synonymous (d_S_, i.e. non-codon changing substitutions) distances for the nucleic acid sequences from all patient and tissue compartments as well as for individual tissue samples. All distances and phylogenetic analyses were performed through pair-wise comparison and pair-wise gap stripping of each sequence.

### Real-time PCR analysis of viral genome and *nef *gene expression in the gut tissues

Total RNA was isolated from the gut tissues using Trizol (Life Technologies, Burlington ON) and used as template for the synthesis of cDNA using a previous described protocol [62,66]. Real-time quantitative PCR using this cDNA and the iCycler IQ system (Bio-Rad, Mississauga, Canada) was performed as follows. The cDNA was diluted 1/1 with water, and 5 μl was used per PCR reaction. Each 25 μl reaction contained 5 μl of cDNA, 11.5 μl of Supermix (Bio-Rad), 6.5 μl of SYBR-Green (1/50,000 dilution; Bio-Rad), 1 μl of fluorescein (1/10,000; Bio-Rad), and 1 μl of primer mix. The amplification protocol consisted of an initial denaturation step of 3 min at 95°C, followed by 45 cycles of 95°C for 30 s, 30 s at the annealing temperature (T_m_) of the primers, and 72°C for 30 s, and a final elongation step of 72°C for 1 min. The amplicons were subjected to a melt curve analysis to ensure proper amplification, in which the temperature was raised from 65 to 99°C in 1°C increments, and data acquired for 8 s at each temperature increment. The viral genome, *nef *containing RNA, and *nef *mRNA levels were normalized against GAPDH (glyceraldehyde-3-phosphate dehydrogenase) and expressed as relative fold change compared to the RNA levels in the esophagus. All real-time RT-PCR quantifications were performed in duplicate and repeated with two different batches of cDNA. The primers used were: *nef *Forward (NEF8748F) 5'-GAA GAA TAA GAC AGG GCT-3', Reverse (NEF8879R) 5'-TCC CAC CCC ATC TGC TGC TGG-3' (Tm = 50°C, 2.5 μM each primer) [62], HIV-1 *gag *Forward 5'-TGC TAT GTC AGT TCC CCT TGG TTC TCT-3', and Reverse 5'-AGT TGG AGG ACA TCA AGC AGC CAT GCA AAT-3' (Tm = 60°C, 2.5 μM each primer)[67], *nef *mRNA Forward 5'-GGC TAA CTA GGG AAC CCA CTG G-3', Reverse 5'-CAG GGA AGT AGC CTT GTG TGT GG-3' (Tm = 50°C, 10 μM each primer) [52], GAPDH Forward 5'-GAA GGT GAA GGT CGG AGT C-3', Reverse 5'-GAA GAT GGT GAT GGG ATT TC-3' (Tm = 50–60°C, 2.5 μM each primer).

### Statistical analyses

All statistical analyses were performed using Graphpad InStat Version 3.01. (GraphPad Software, San Diego, CA) and *p *values < 0.05 were considered significant.

## Competing interests

The author(s) declare that they have no competing interests.

## Authors' contributions

GvM, MJG, and DLC collected and analyzed data, designed the study, recruited patients and were involved in writing the paper. DK, LM and TG were involved in designing the methods for collecting, analyzing and interpreting data, and helped putting the data and parts of the manuscript together for publication.
